# Cardiovascular Risks of Ketogenic Diet for Glut-1 Deficiency

**DOI:** 10.15844/pedneurbriefs-32-8

**Published:** 2018-09-05

**Authors:** Alberto Spalice, Cristiana A. Guido

**Affiliations:** 1 Department of Pediatrics, Child Neurology Division, Sapienza University of Rome, Rome, Italy

**Keywords:** Glut-1 Deficiency, Ketogenic Diet, Cardiovascular Risk Factors

## Abstract

Investigators from Children's Hospitals of Aschaffenburg-Alzenau, Germany, and Essen University, Germany reported on long-term cardiovascular risks of ketogenic diet (KD) treatment for Glut1 Deficiency syndrome (GLUT1-DS).

Investigators from Children's Hospitals of Aschaffenburg-Alzenau, Germany, and Essen University, Germany reported on long-term cardiovascular risks of ketogenic diet (KD) treatment for Glut1 Deficiency syndrome (GLUT1-DS). Ten patients were followed for 10 years as part of a prospective study with monitoring of body mass index, cholesterol, and triglycerides at baseline, then 6 months, 2 years, 5 years, and 10 years following initiation of the KD. At ten years, the cardiovascular risk was assessed by body mass index, carotid intima-media thickness, and blood pressure (using healthy reference population as a control). Results may be summarized as no significant differences in body mass index, cholesterol, or blood pressure after 10 years on the KD. Although previously published studies of short-term follow-up of adverse effects of KD showed possible cardiovascular risks, this study with a longer follow up duration did not. [1]

COMMENTARY. Transport of glucose from the bloodstream across the blood-brain barrier to the central nervous system is facilitated by glucose transport protein type 1, the first member of the solute carrier family 2. Heterozygous pathogenic variants in the *SLC2A1* gene cause cerebral energy failure and a symptoms referred to as GLUT1-DS. Clinical features usually comprise motor and mental developmental delay, seizures with infantile onset, deceleration of head growth often resulting in acquired microcephaly, and a movement disorder with ataxia, dystonia, and spasticity. Subsequent to the delineation of this classic phenotype the variability of signs and symptoms in GLUT1-DS is being recognized. Patients with (i) carbohydrate-responsive symptoms, with (ii) predominant ataxia or dystonia, but without seizures, and with (iii) paroxysmal exertion-induced dyskinesia and seizures have been reported. Common laboratory hallmark in all phenotypes is the reduced glucose level in cerebrospinal fluid with lowered CSF-to-blood glucose ratio. Treatment with a KD results in marked improvement of seizures and movement disorders [2].

The risk benefit of KD in pediatric epileptic patients is still controversial, even if some benefits are absolutely available especially in GLUT1-DS. Seizures and cognitive functions were greatly improved with KD treatment, but less effective for the other neurological disorders of the patients. Alternatives dietary therapies may be helpful when the classic KD is not tolerated [3].

The effects of a long-term KD in children with Glut1 deficiency syndrome on metabolism are unknown. Our results indicate a characteristic effect of a long-term KD on glucose and lipid homeostasis in Glut1 deficiency syndrome. Although serum lipids and apolipoproteins reflect a proatherogenic lipoprotein profile, adipocytokine constellation is not indicative of enhanced cardiovascular risk [4].

In conclusion, long-term follow-up on cardiovascular risk of KD refutes prior reports. In order to evaluate cardiovascular risks at least 5 year follow-up is mandatory. Initial dyslipidaemia resolves over time and remains normal at 10 years while carotid intima-media thickness does not increase during long-term application. KD remains the treatment of choice for GLUT1-DS [1].

## Disclosures

The author(s) have declared that no competing interests exist.
